# Trajectories of Antidepressant Medication before and after the Onset of Unemployment by Subsequent Employment Experience

**DOI:** 10.1371/journal.pone.0169652

**Published:** 2017-01-05

**Authors:** Taina Leinonen, Netta Mäki, Pekka Martikainen

**Affiliations:** 1 Population Research Unit, Department of Social Research, University of Helsinki, Helsinki, Finland; 2 City of Helsinki Urban Facts, Helsinki, Finland; 3 Centre for Health Equity Studies (CHESS), Stockholm University and Karolinska Institutet, Stockholm, Sweden; 4 Max Planck Institute for Demographic Research, Rostock, Germany; Stellenbosch University, SOUTH AFRICA

## Abstract

**Background:**

The unemployed more often suffer from depression than the employed. We examined whether mental health deterioration occurs already before unemployment implicating health selection, or whether it mostly occurs after becoming exposed to the experience rendering causal explanations more likely.

**Methods:**

We used nationally representative Finnish register data to examine changes in depressive morbidity as measured by antidepressant medication in 1995–2009 over four years before and since a new onset of unemployment (N = 28 000) at the age of 30–60 compared to the employed (N = 124 136). We examined separately those who became continuously long-term unemployed, intermittently unemployed and unemployed with eventual re-employment in the second, third or fourth year since the year of onset. Annual repeated measurements were analysed using generalised estimation equations.

**Results:**

Among the employed antidepressant medication increased slowly but steadily over the study period and it was mainly at a lower level than among the unemployed. In the four years leading to unemployment there was excess increase in medication that was generally stronger among those with longer duration of the eventual unemployment experience. During unemployment medication decreased in all groups except among the intermittently unemployed. By the first year of re-employment antidepressant medication reached a level similar to that among the employed and afterwards followed no consistent trend.

**Conclusions:**

The associations of unemployment and re-employment with depressive morbidity appear to be largely driven by health selection. The question of potential causal associations remains unresolved for intermittent unemployment in particular.

## Introduction

Those who are unemployed are more likely to suffer from depression and other mental health problems than the employed [1–3]. This may be caused by selection of those with health problems into unemployment or by causal negative health effects of job loss and unemployment [4]. Previous studies including meta-analyses have found support for both mechanisms. On the one hand pre-existing mental ill health is associated with an increased risk of unemployment [2, 5–8] and a lower likelihood of re-employment [2, 9, 10]. On the other hand unemployment is followed by poorer [1–3, 6, 11, 12] and re-employment by improved mental health outcomes [1, 2, 9, 12, 13].

A study based on Australian data from the 2000s has simultaneously tested the reciprocal associations using cross-lagged path analysis and found that mental ill health is a stronger predictor of unemployment than vice versa [6]. Other recent studies from Norway [14] and Sweden [15] have addressed the direction of the association by examining trajectories of psychotropic medication before and after the onset of unemployment. Overall, these studies suggest that in addition to health selection, at least short-term effects of unemployment on mental ill health are likely. However, the largest effect appeared to occur at a time of anticipation of job loss instead of after the onset of unemployment [14]. Furthermore, a mental health decline was observed among individuals experiencing downsizing more generally, and not solely among those who subsequently become unemployed [15]. The effects of unemployment on mental ill health may thus be largely related to overall experiences of job insecurity and less to the particular status of actually being without a job.

On the other hand, previous studies suggest that the association with mental ill health becomes stronger with longer duration of unemployment [1, 2, 11, 14] and with recurrent or cumulative exposures [16–19]. Other studies have, however, found that mental ill health after job loss and unemployment does not necessarily increase with recurrent exposures among the initially employed [20] or among older adults approaching retirement age [21]. The association between unemployment and mental ill health may also vary by socio-demographic factors, although findings have been inconsistent. A meta-analysis found that unemployment was followed by worse outcomes among men, but there is little consistent variation by age, socioeconomic position and marital status [2].

Overall, the role of health selection and causation is still unclear with respect to the association between unemployment and mental ill health. Little is known of patterns of long-term changes in mental ill health and recovery over several years before and after unemployment and re-employment. We examined trajectories of depressive morbidity using annual repeated measurements over four years before and since a new onset of unemployment in order to evaluate whether mental health deterioration tends to occur already before the experience of unemployment, or whether it mostly occurs after becoming exposed. We also assessed the influences of duration of unemployment and eventual re-employment as well as the modifying effects of socio-demographic factors.

## Material and Methods

### Study population and measurement of unemployment

We used administrative register data linked by Statistics Finland by means of unique personal identification numbers. This study was based on a longitudinal data set for which Statistics Finland has drawn a nationally representative 11% sample of the total population permanently residing in Finland at the end of any of the years 1987–2007. In addition, those who died were oversampled to cover 80% of all deaths occurring in Finland during that period. Because of the different sampling probabilities among the deceased and the living, analytical weights were used in the analyses. The data include annual information on the number of months employed and unemployed, purchases of antidepressant medication and socio-demographic factors until the end of 2009.

**Fig 1:**
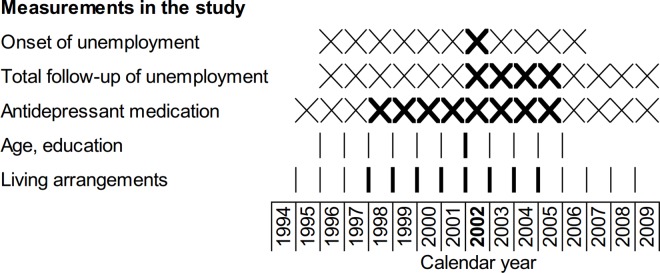
Calendar years for key measurements in the study. Crossing lines indicate information for the whole year and the vertical lines for the end of year status; as an example the measurement years for those whose first onset of unemployment was in 2002 are shown in bold.

Information on unemployment was based on the annual number of months a study person was registered as a jobseeker which is required in Finland for the receipt of unemployment benefits. We defined being unemployed as having more than one month of unemployment and being employed as having at least eleven months of employment in a particular calendar year. The one-month limit for defining unemployment was chosen in order to exclude short breaks in employment that may be related to job change. We included those aged 30–60 at their first onset of unemployment between 1996 and 2006. We were interested in new episodes of unemployment in order to better assess the health selection process. Those who had experienced more than one month of unemployment per year in the preceding four-year period (e.g. those whose year of onset was 1996 and who had experienced unemployment in 1992–1995) were therefore excluded (55.4% of the original unemployed group). Duration of the unemployment experience and re-employment were followed up over a maximum of four years, the calendar periods thus ranging between 1996–1999 and 2006–2009 for a single study person (1996–1999, 1997–2000, 1998–2001, …, 2006–2009). The reference population included people from the same cohorts who were employed during one of these randomly selected four-year periods and who had experienced no more than one month of unemployment per year in the preceding four-year period. For the employed the onset year of unemployment thus corresponds to a randomly selected year between 1996 and 2006. Calendar years for key measurements in the study are presented in Fig 1.

We excluded those who were not employed in the year preceding the onset of unemployment (33.7% among the unemployed and 3.5% among the employed), those who retired by the end of the year of onset (0.3% and 0.2%) and those who immigrated in the four years before (7.8% and 0.4%) or emigrated in the four years since the year of onset (0.2% and 0.2%). The study population consisted of 28 000 unemployed and 124 136 employed persons.

Those who became fully re-employed after the initial onset year of unemployment, i.e. were employed annually for at least 11 months since the second, third or fourth year of the unemployment follow-up, were examined separately from those with no re-employment. The latter group was further divided into two: those who were annually unemployed for 12 months after the initial year of onset were defined as continuously long-term unemployed and the rest as intermittently unemployed. Among the intermittently unemployed 90% were employed at least once during the four-year follow-up with an average of 3.8 months of annual employment. Thus the employment categories were: a) continuously long-term unemployed, b) intermittently unemployed, c) unemployed, re-employment 2nd year, d) unemployed, re-employment 3rd year and e) unemployed, re-employment 4th year and f) employed (reference). Because our definition of unemployment is based on having more than one month of unemployment in a particular calendar year, time before re-employment does not necessarily indicate the duration of a single unemployment episode, but rather the duration of experiencing one or more episodes of unemployment before full re-employment. After the onset year of unemployment, a study person was censored in the year of retirement, other labour force exit or death. A re-employed study person was censured in the year of recurring unemployment. Labour force exit including retirement was the main reason for censoring (92.2%). Altogether 11.6% of the employed and 42.3% of the unemployed were censored. Among the unemployed, censored individuals were more often categorized as continuously long-term unemployed (36.9% among the censored and 13.3% among the non-censored) or intermittently unemployed (44.6% and 33.8%) and consequently less often as re-employed.

### Measurement of depressive morbidity and socio-demographic factors

Antidepressant medication over a maximum of four years before and four years since the onset year of unemployment was used as the dependent variable. Although antidepressants are prescribed by a physician for conditions requiring treatment, their use is not a direct measure of diagnosed depression. In this study we consider antidepressant medication as an indicator of depressive morbidity, but it should be noted that the measure does not fully or solely capture the population suffering from depression (see the Methodological considerations chapter for more details). Medication data was available for the period 1995–2009. As the earliest year of onset was 1996, antidepressant medication was observed for at least one year before unemployment for each person. The measure was based on complete information on annually purchased defined daily doses (DDD) of prescribed medication among the study population obtained from the reimbursement register of the Social Insurance Institution of Finland. Antidepressants were identified using the Anatomical Therapeutic Chemical (ATC) classification code N06A.

We used socio-demographic factors as control variables and potential effect modifiers. Age and education were measured at the end of the year preceding the onset year of unemployment. Age was categorised into five-year groups and in the interaction analyses to ten-year groups. Education was based on the highest completed level and was categorised as 1) primary (up to 9 years), 2) secondary (up to 12 years) and 3) tertiary education (13+ years). The secondary and tertiary groups were combined in the interaction analyses. Living arrangements included household information on the number and family relations of the residents. The categories used were living 1) alone, 2) with a partner only (marital and non-marital cohabitation), 3) with a partner and children (own or partner’s children), 4) with children only (single parent), 5) with other people and 6) other (does not belong to the household population or unknown). Living arrangements were used as time-varying covariates with information updated at the end of each calendar year. In the interaction analyses we examined those living with (groups 2 and 3) or without a partner (groups 1, 4 and 5) at the end of the year preceding the onset year of unemployment.

The unemployed who were excluded (mainly because of previous unemployment) and those remaining in the final study population had similar gender distributions, but the groups somewhat differed by other socio-demographic characteristics. The excluded were younger (mean age 42.9 among the excluded versus 46.6 among the included), more likely to have basic education (39.0% versus 35.0%), and more likely to live without a partner (34.9% versus 28.7%).

### Statistical methods

The analyses of mean DDD of antidepressant medication were based on linear regression using generalised estimation equations (GEE). GEEs account for the interdependence between repeated within-subject measurements by assigning them a correlation structure [22]. An autoregressive correlation structure was chosen on the assumption that the correlation is stronger between observations that are closer to each other in time. Associations of unemployment and re-employment with changes in mean DDD were assessed by examining interactions between employment category and study year, i.e. the years before and since the onset of unemployment. Study year was used both as categorical and continuous variables. With the former, graphical trajectories were plotted using estimated marginal means, thereby showing the predicted mean DDD in each year for each employment category averaged over the other covariates. With the latter, excess annual changes in mean DDD among the unemployed groups compared to the employed were calculated in time frames in relation to the onset of unemployment and re-employment. We also tested whether socio-demographic factors modified these excess annual changes during unemployment. All models were adjusted for age, gender, education, living arrangements and calendar year. Adjustment for calendar year accounts for the increasing secular trend in the prescription of antidepressants.

We chose to use DDDs instead of a dichotomous measure of any antidepressant medication because of the long time intervals based on calendar years, during which the amount of medication purchased varies considerably between individuals. We nevertheless performed sensitivity analyses calculating the predicted annual percentage of any purchases of antidepressant medication based on marginal means derived from a GEE model applying repeated logistic regression. We also performed sensitivity analyses for the predicted annual mean DDD excluding those who were censored after the onset of unemployment.

### Ethics statement

We followed data protection guidelines and ethical regulations approved by the data protection authorities, Statistics Finland and the University of Helsinki in the collection, use and reporting of the data. Statistics Finland provided permission to use the anonymous register-based data.

## Results

Over a third of the unemployed were eventually re-employed (Table 1). The unemployed had a higher percentage of any antidepressant medication during the study years (17%) than the employed (12%). Medication was more common among the intermittently unemployed than the continuously long-term unemployed. Longer duration of the unemployment experience before re-employment was also associated with a higher level of medication. Those who became re-employed were younger and better educated than the other unemployed groups. Of those who were continuously long-term unemployed 95% were 50+ years and 53% had only primary education.

**Table 1:** Number of study participants (N), types of unemployment experience (%), purchases of any antidepressant medication in the study period (%) and socio-demographic factors (%) by employment categories.

			Unemployed					
		Employed	Continuous long-term	Inter-mittent	Re-em-ployment 2nd year	Re-em-ployment 3rd year	Re-em-ployment 4th year	Total
Unweighed N		124 136	6 913	11 404	5 643	2 711	1 329	28 000
Unemployment types, %		–	23.3	38.4	22.3	10.8	5.3	100.0
Any antidepressants								
	Yes	11.7	12.6	21.3	12.7	15.3	17.4	16.5
	No	88.3	87.4	78.7	87.3	84.7	82.6	83.5
Gender								
	Men	52.4	49.8	45.7	57.1	50.3	45.6	49.7
	Women	47.6	50.2	54.3	42.9	49.7	54.4	50.3
Age								
	30–34	12.2	0.6	13.4	20.2	17.9	15.7	12.6
	35–39	16.4	1.1	14.2	20.9	19.8	20.5	13.6
	40–44	19.4	1.3	16.7	20.1	21.9	22.1	14.7
	45–49	20.4	2.1	19.0	18.9	21.0	23.1	15.5
	50–54	18.4	15.3	20.9	14.6	14.6	14.2	17.2
	55–60	13.1	79.4	15.8	5.3	4.7	4.4	26.5
Education								
	Primary	21.5	53.3	34.1	22.9	26.9	28.3	35.0
	Secondary	39.9	29.8	42.5	46.6	44.5	43.5	40.7
	Tertiary	38.7	16.9	23.4	30.5	28.6	28.2	24.3
Living arrangements								
	Alone	13.1	17.6	18.9	15.2	15.7	16.2	17.2
	With partner only	23.0	52.8	27.5	20.1	19.9	20.0	29.8
	With partner & children	54.4	22.5	39.9	53.0	51.7	50.9	41.4
	With children only	5.3	3.0	6.9	5.4	6.7	6.9	5.7
	With other people	3.8	3.8	6.2	5.7	5.4	5.2	5.4
	Other	0.3	0.3	0.7	0.5	0.7	0.9	0.6
Total, %		100.0	100.0	100.0	100.0	100.0	100.0	100.0

**Fig 2:**
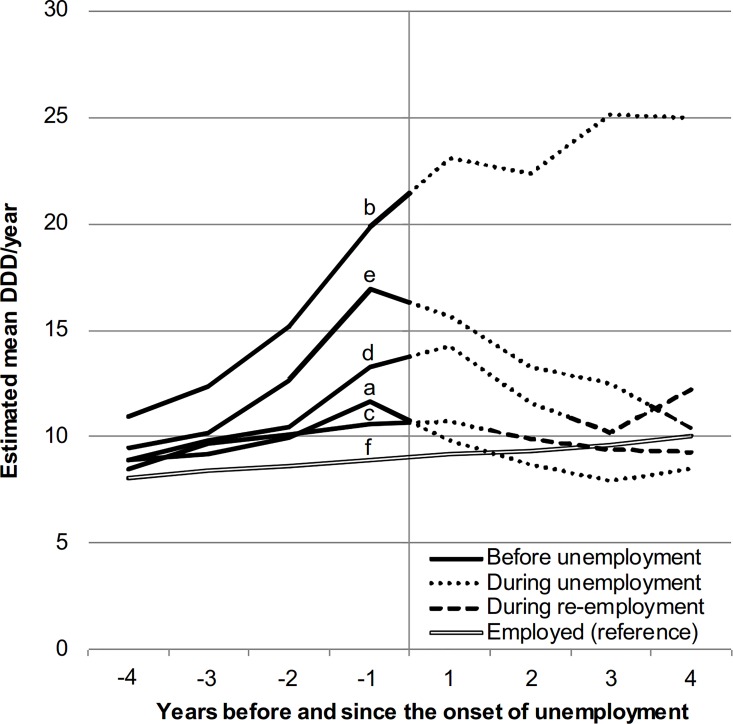
Trajectories of antidepressant medication before and since the onset of unemployment. Estimated mean DDD/year among those with (a) continuous long-term unemployment, (b) intermittent unemployment, (c) re-employment in the second (d) third or (e) fourth year since the year of onset, as well as (f) the employed reference group. Adjusted for age, gender, education, living arrangements, and calendar year.

Fig 2 presents the predicted mean DDD in each of the study years which may reflect time either before unemployment, during unemployment, or during re-employment (and in the corresponding years for the employed reference group). Allocation of the study years to these statuses varies according to the previously constructed six employment categories for which the employment transitions by definition occur in the same years. Among the employed antidepressant medication increased slowly but steadily over the study years (Fig 2F). The level of medication was constantly highest among those who became intermittently unemployed (b). Among this group medication increased substantially in the four years leading to unemployment after which the increase continued more slowly. Among those who became continuously long-term unemployed (a) and those who eventually became re-employed (c–e) the level of antidepressant medication four years before unemployment was relatively close to that among the employed. However, in the year before the onset of unemployment, it had risen to much higher levels, particularly among those with two or three years of eventual unemployment before re-employment. Medication decreased during unemployment among both the continuously long-term unemployed and those who eventually became re-employed. Among the former, the decrease was clearly beyond the level of the employed. Among the latter, medication reached a similar level than among the employed by the first year of re-employment.

Excess changes in antidepressant medication over time in relation to unemployment and re-employment are presented in Table 2. The coefficients express the annual change in the mean DDD exceeding that found among the employed reference group. Excess increase in medication before unemployment was statistically significant for all groups except those who eventually became re-employed in the second year. Over the transition from employment to unemployment, there was a decrease in medication among those who became continuously long-term unemployed, an increase among those who became intermittently unemployed and no change among those who eventually became re-employed. During unemployment medication decreased in all groups except among the intermittently unemployed. Medication also tended to decrease over the transition from unemployment to re-employment. During re-employment there were no statistically significant changes in medication. However, when combining the time frames corresponding to the transition to and time during re-employment in the second year, a significant excess decrease (-0.80, 95% CI -1.36–-0.25) was found.

**Table 2:** Excess annual change in mean DDD of antidepressant medication compared to the employed reference group and 95% confidence intervals (CI) in time frames in relation to unemployment and re-employment.

	Time frames									
	Before unemployment		Transition to unemployment^b^		During unemployment		Transition to re-employment^c^		During re-employment	
Unemployment	b^a^	(95% CI)	b^a^	(95% CI)	b^a^	(95% CI)	b^a^	(95% CI)	b^a^	(95% CI)
Continuous long-term	0.67	(0.12–1.23)	-2.10	(-3.26–-0.93)	-0.93	(-1.60–-0.25)	–	–	–	–
Intermittent	2.80	(2.22–3.37)	3.04	(1.66–4.41)	0.37	(-0.47–1.21)	–	–	–	–
Re-employment 2nd year	0.41	(-0.10–0.92)	-0.18	(-1.33–0.97)	–	–	-1.02	(-2.15–0.10)	-0.67	(-1.45–0.11)
Re-employment 3rd year	1.23	(0.33–2.13)	0.74	(-1.74–3.21)	-2.79	(-5.19–-0.40)	-1.68	(-3.42–0.06)	1.62	(-0.80–4.05)
Re-employment 4th year	2.32	(0.60–4.04)	-1.45	(-4.49–1.60)	-1.80	(-3.70–0.10)	-2.46	(-4.73–-0.19)	–	–

Adjusted for age, gender, education, living arrangements and calendar year.

^a^Coefficient for the annual change exceeding that of the employed reference group, for which there was an increase of 0.29 DDDs per study year across all time frames.

^b^Change between the last year of employment and the first year of unemployment.

^c^Change between the last year of unemployment and the first year of re-employment.

We systematically assessed for the interactions between socio-demographic factors and changes in antidepressant medication during unemployment; with the only significant association found for gender among the intermittently and continuously long-term unemployed (S1 Fig). The decrease in medication during continuous long-term unemployment was stronger among women. Furthermore, medication continued to increase during intermittent unemployment only among men.

The shape of the trajectories was very similar in the sensitivity analyses excluding those who were censored after the onset of unemployment (S2 Fig). There were, however, some differences in the levels: antidepressant medication was higher among the continuously long-term unemployed and lower among the intermittently unemployed compared to the levels of these groups in the full sample. The trajectories were also relatively similar in the sensitivity analyses where any antidepressant medication was used as the outcome instead of the DDDs (S3 Fig). The main difference was that for the intermittently unemployed there was no increasing trend in antidepressant medication during unemployment.

## Discussion

### Health selection and causation

We used repeated measurements of annual mean DDD of antidepressant medication to examine depressive morbidity over several years around a new onset of unemployment while accounting for duration of unemployment and potential re-employment. Even though the methodology did not allow for a quantitative testing of the relative contributions of health selection and causation, the direction of the association between employment transitions and depressive morbidity could be assessed indirectly.

The findings indicate that depressive morbidity is more likely to be followed by unemployment than vice versa. Morbidity increases in the years leading to unemployment, particularly in cases of intermittent episodes or long duration before eventual re-employment. By way of exception, such health decline is not particularly strong for those eventually becoming continuously long-term unemployed over several years. Nevertheless, all of the unemployed groups have poorer mental health already before becoming exposed. Even though in Finland employees are not allowed to be dismissed on health grounds, those with poorer health may be among the first to be made redundant and have poorer employment opportunities after termination of temporary contracts. We found that the observed increases in depressive morbidity mostly restrict to the time before unemployment. Even though morbidity continues to increase since transition to intermittent unemployment, this happens at a slower pace than in the preceding years, therefore more likely to reflect continuance of an already declining trend in mental health than a negative outcome triggered by unemployment.

Comparing our study and the two previous ones that have examined trajectories of antidepressant medication before and after the onset of unemployment is difficult due to the different study designs used, e.g. the categorization of unemployment by its duration and by potential re-employment status in our study, shorter time intervals and a measure of incident purchases of medication in the Norwegian study [14], and a downsizing context in the Swedish study [15]. However, the Norwegian findings indicate that the risk of incident purchases of antidepressants and other psychotropic medication increased over the last three months of the examined six-month period before the onset of unemployment, peaked one month before unemployment, and decreased at least over the first three months during unemployment. Over the examined six-month period after re-employment the risk was at a similar level as six months before the onset of unemployment. The Swedish findings indicate that for initially healthier employees the prevalence of purchases of antidepressants increased between one year before and one year after major workplace downsizing. This change occurred not only among those who became unemployed, but among remaining employees and job changers as well. For those with poorer initial health status, the prevalence actually decreased around the time of downsizing among all three groups. Over both the pre-and post-downsizing periods the prevalence of antidepressant medication was nevertheless at a much higher level among those who became unemployed than among the other groups, suggesting selection of those with poorer health into unemployment in the situation of downsizing. Thus, there seems to be no large controversies between these findings and ours.

Previous research suggests that the association between mental ill health and unemployment is stronger among men [2, 11, 14, 23]. The present study indicates that the relative developments in depressive morbidity during continuous long-term and intermittent unemployment are more unfavourable among men. Even among men, however, the increase in morbidity is larger before than during unemployment.

Our findings also indicate that improvement in depressive morbidity is more likely to be a prerequisite for re-employment than a positive effect related to termination of the unemployment exposure and return to work. Firstly, a large part of the health improvement occurs while still experiencing unemployment, depressive morbidity having already decreased down to a similar level than among the employed population by the time of re-employment. Secondly, there is no consistent further improvement during re-employment. The modest decrease in depressive morbidity among those who become re-employed in the year immediately after the onset of unemployment is therefore not likely to be attributable to any general effects of re-employment, but rather to particular circumstances among this group, for which almost 60% of the unemployment experiences did not exceed three months (result not shown in the tables). Re-gaining employment quickly may, for example, reflect better employment prospects and access to better quality jobs. Mental health may be influenced not solely by gaining employment but also by the quality of the attained job [24, 25].

All in all, the results of the present study appear to support health selection more than causal associations of unemployment and re-employment with depressive morbidity. Previous studies on mental health have reached mixed findings on the role of these two mechanisms [6, 9, 12, 26–28]. Both selection and causation also receive support when examining the association between unemployment and suicide [29–31], an outcome closely related to mental ill health. Previous findings from Finland nevertheless suggest that the association between unemployment and all-cause mortality may be largely attributable to selection effects [32]. Causal effects of unemployment on ill health may be smaller in countries with generous social security systems such as Finland. A recent study found worsening trajectories of self-rated health during unemployment in all other European welfare regimes than the Nordic region [33]. Studies that do not properly account for health selection may reach misleading conclusions on the consequences of unemployment. The selection effect may be underestimated if, for example, health is measured only at one point before unemployment [34]. Moreover, our findings based on long-term trajectories suggest that the association between unemployment and mental ill health may be largely driven by health selection even if morbidity were constantly at a higher level during than before unemployment.

### Duration of unemployment

Our study design with retrospectively defined employment categories allowed for assessment of trajectories of depressive morbidity among groups with particular unemployment and re-employment experience. The purpose of this study was therefore not to estimate and make inference about the effects of depressive morbidity on the duration of unemployment or on re-employment. Our findings indicate that trajectories of depressive morbidity vary considerably by whether unemployment is continuous and long-term or intermittent. Previous research indicates that the association with mental ill health is stronger with longer duration of unemployment [1, 2, 11, 14] and with recurrent or cumulative experiences [16–19]. However, linear associations with duration may only apply to episodes lasting for less than a year [2], making comparisons with the present study difficult. Our findings suggest that an increase in depressive morbidity is connected less with continuous long-term than intermittent unemployment, the latter reflecting prolonged unstable labour market positions typically including successive periods of unemployment and employment. Although health selection is likely to play a role in the process, stress related to labour market instability and associated precarious employment may also contribute [35, 36]. Previous findings suggest that a history of prolonged unemployment and job insecurity are jointly associated with a subsequent higher use of antidepressants [37]. It should, however, be noted that the present study only includes new episodes of unemployment and therefore does not provide information on mental health trajectories among those with constant unemployment experiences in the past.

We found that those who became continuously long-term unemployed are a special group consisting of older and less educated people whose trajectory of depressive morbidity did not differ to a great extent from that of the employed population. The finding is unlikely to be explained by straightforward influences of age and education, since no interactions were found by these factors among the intermittently unemployed. Our conclusions may nevertheless be inapplicable to younger or more educated groups which could not be analysed separately among the continuously long-term unemployed due to their small numbers. The continuously long-term unemployed may have been more often subject to structural unemployment that is unlikely to be influenced by health and other individual characteristics. Previous research suggests that the association between unemployment and health is more modest when external factors contribute to the transition such as in contexts of high levels of unemployment, rapid downsizing or workplace closure [23, 28, 32]. The observed decline in antidepressant medication during continuous long-term unemployment may be partly due to a decrease in depressive symptoms, smaller need for treatment due to the removal of work-related psychosocial demands or poorer access to treatment (see the Methodological considerations -section for more discussion on access to treatment).

### Methodological considerations

We used a large nationally representative population sample based on longitudinal register data with no self-reporting bias or loss to follow-up. One major advantage was the design based on repeated measurements of morbidity and employment status. Examination of long-term health trajectories over several years around unemployment and re-employment helps to assess whether changes in health mainly occur before or after these transitions.

One shortcoming was the lack of exact dates for unemployment episodes. Those who had several months of both unemployment and employment in a particular calendar year were defined as unemployed. As a result, the reported decline in medication during unemployment may have partly taken place while already transitioned to re-employment, because this may have begun in the preceding calendar year. This is, however, unlikely to have largely influenced our conclusions because the decline was observed already three years before re-employment.

Using antidepressant medication as a measure of depressive morbidity has certain weaknesses. Antidepressant use does not directly equate with diagnosed depression among the study population. The measure captures only conditions treated with medication. A large proportion of those with depression do not receive treatment [38]. The slower increase and even decrease in medication during unemployment in the present study may be partly explained by a larger unmet need for health care among the unemployed [39–41], particularly the long-term unemployed [39]. Despite partial reimbursement of drugs including antidepressants, cost is still a common barrier to the use of prescription medicines in Finland particularly among those with low income and poor health [42]. Other Finnish findings nevertheless suggest that employment status is not associated with the use of antidepressants among those with major depressive disorder [38]. However, especially the observed decline in antidepressant medication among the continuously long-term unemployed is likely to be partly explained by under-treatment of depression as a result of financial difficulties, lack of motivation due to poor chances of re-employment or other disadvantages related to prolonged exclusion from employment. The data also lacked diagnoses for antidepressant prescription. Although antidepressants are primarily used to treat depression, they are also used for conditions such as anxiety, chronic pain and sleep problems [43, 44]. However, non-psychiatric indications are typically more common in older age [43]. Findings from Finland indicate that the majority of antidepressant users aged 40–64 are either currently depressed or have a history of doctor diagnosed depression [45]. Even so, the amount of antidepressants purchased does not necessarily reflect the severity of the treated condition. The use of DDDs as the outcome measure is nevertheless unlikely to lead to misleading results, since we would draw similar conclusions from the analyses irrespective of whether DDDs or a dichotomous measure of any purchases of antidepressants were used. Finally, it should be kept in mind that antidepressant treatment may have curative effects that reduce the likelihood of becoming unemployed. This may lead to underestimation of the magnitude of increased depressive morbidity before unemployment, but is unlikely to influence our general conclusions.

Since our analyses were based on the population-averaged GEE-model, the shape of the trajectories reflects changes in the mean level of antidepressant medication among a particular employment category. Individual trajectories within these categories may largely vary. For example, a stable mean trajectory may signify a constant level of medication among some individuals, but at the same time, some medication users may become non-users and vice versa. Measuring duration of use or identifying typical patterns of use around employment transitions may be relevant questions for further research, but they are beyond the aims of this study. A further consideration relates to a skewed distribution of DDDs due to a large proportion of non-users of antidepressants. Given the large sample, however, it is likely that the mean DDD will be normally distributed around the true population mean despite the non-normal distribution of the variable, thus resulting in a valid estimation of standard errors [46].

The increase in depressive morbidity before unemployment and the decrease before re-employment can be partly attributable to anticipatory effects. Previous findings on the effects of anticipation of job loss on mental ill health have been inconsistent [14, 35, 47]. Norwegian findings nevertheless indicate that the largest increase in the risk of incident purchases of antidepressants occurs in the three months before the onset of unemployment [14]. Much of the potential anticipatory effect in our present study is thus likely to occur in the year of onset of unemployment instead of the preceding years. While expecting particular unemployment events several years in advance is unlikely, experiences of overall job insecurity, unfavourable work environment and other labour market disadvantages may be more common among those who eventually become unemployed. During unemployment the observed changes, i.e. more modest increase than before or even decrease in morbidity, may be related to recovery among some individuals after a depressive episode that was originally associated with becoming unemployed. The normal course of depression often consists of periods of recovery and recurrence [48].

We found that censoring of some individuals after the onset of unemployment—mainly due to labour force exit—had little effect on the shape of the trajectories of antidepressant medication and therefore did not influence our general conclusions. After excluding those who were censored, the overall level of antidepressant medication nevertheless increased among the continuously long-term unemployed and decreased among the intermittently unemployed. Labour force exit thus seems to be associated with being better off among the continuously long-term unemployed and with being worse off among the intermittently unemployed. Consisting of older individuals who are closer to typical retirement ages, the continuously long-term unemployed who exit the labour market may often take a more “normal” retirement pathway, whereas the intermittently unemployed who exit the labour market typically do so prematurely through pathways including work disability.

## Conclusions

In these data, the association of unemployment and re-employment with depressive morbidity seems to be largely driven by health selection. Misleading conclusions may be drawn about health effects of unemployment on the basis of studies that do not properly account for selection effects. Furthermore, the trajectories of depressive morbidity crucially depend on the duration of unemployment and on subsequent re-employment. The question of potential causal associations remains unresolved for those experiencing intermittent unemployment in particular.

## Supporting Information

S1 Fig Trajectories of antidepressant medication before and since the onset of unemployment by gender. Estimated mean DDD/year among (A) men and (B) women with (a) continuous long-term unemployment, (b) intermittent unemployment, as well as (c) the employed reference group. Adjusted for age, education, living arrangements, and calendar year. Three-way interaction between gender, belonging to the unemployed group (as opposed to being employed), and a continuous study year variable during (a) continuous long-term unemployment: p = 0.044 and (b) intermittent unemployment: p = 0.029.
(PDF)

S2 Fig Trajectories of antidepressant medication before and since the onset of unemployment excluding those who were censored after the onset of unemployment. Estimated mean DDD/year among those with (a) continuous long-term unemployment, (b) intermittent unemployment, (c) re-employment in the second (d) third or (e) fourth year since the year of onset, as well as (f) the employed reference group. Adjusted for age, gender, education, living arrangements, and calendar year.
(PDF)

S3 Fig Trajectories of antidepressant medication before and since the onset of unemployment using a dichotomous outcome measure. Estimated percentage of any purchases of antidepressant medication per year among those with (a) continuous long-term unemployment, (b) intermittent unemployment, (c) re-employment in the second (d) third or (e) fourth year since the year of onset, as well as (f) the employed reference group. Adjusted for age, gender, education, living arrangements, and calendar year.
(PDF)
