# Supplementary material for: Trajectories of Antidepressant Medication before and after the Onset of Unemployment by Subsequent Employment Experience
Source: PLoS One. 2017 Jan 5;12(1):e0169652. doi: 10.1371/journal.pone.0169652 (PMC5215907; doi:10.1371/journal.pone.0169652)
Supplement: S1 Fig — Estimated mean DDD/year among (A) men and (B) women with (a) continuous long-term unemployment, (b) intermittent unemployment, as well as (c) the employed reference group. Adjusted for age, education, living arrangements, and calendar year. Three-way interaction between gender, belonging to the unemployed group (as opposed to being employed), and a continuous study year variable during (a) continuous long-term unemployment: p = 0.044 and (b) intermittent unemployment: p = 0.029. (PDF) [file pone.0169652.s001.pdf]

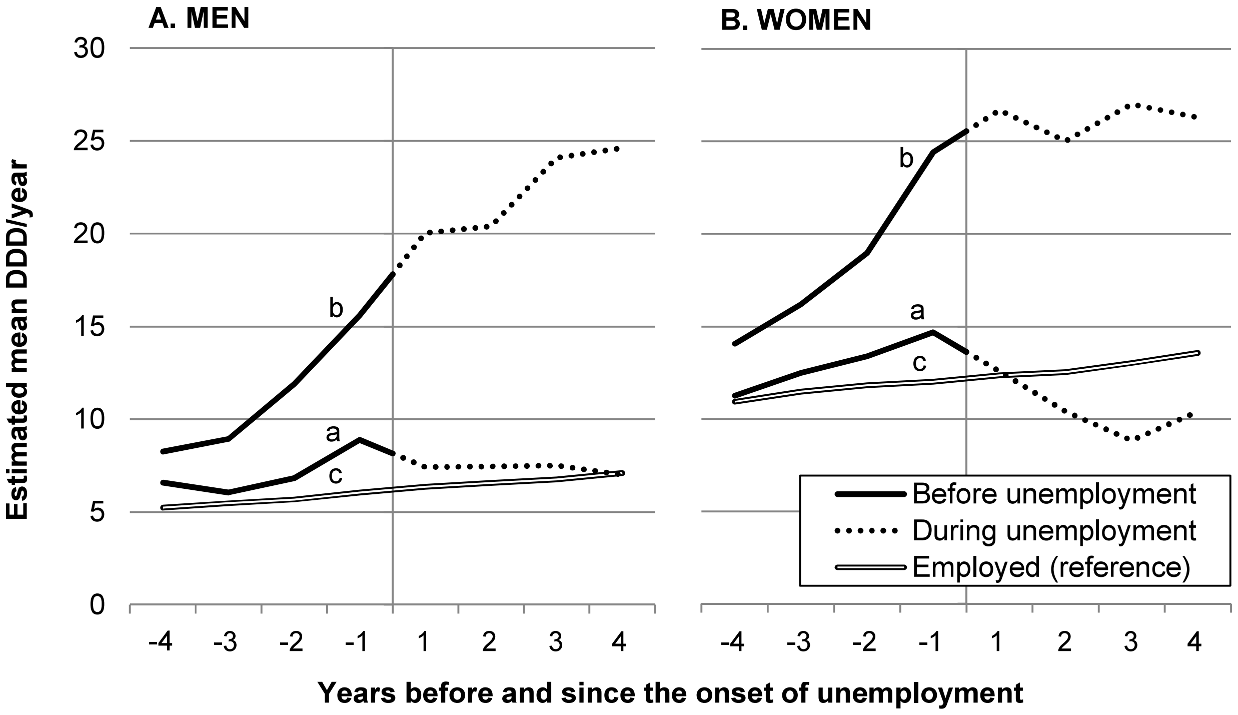

S1 Fig. Trajectories of antidepressant medication before and since the onset of unemployment by gender. Estimated mean DDD/year among (A) men and (B) women with (a) continuous long-term unemployment, (b) intermittent unemployment, as well as (c) the employed reference group. Adjusted for age, education, living arrangements, and calendar year. Three-way interaction between gender, belonging to the unemployed group (as opposed to being employed), and a continuous study year variable during (a) continuous long-term unemployment: p=0.044 and (b) intermittent unemployment: p=0.029.
