# Supplementary material for: Trajectories of Antidepressant Medication before and after the Onset of Unemployment by Subsequent Employment Experience
Source: PLoS One. 2017 Jan 5;12(1):e0169652. doi: 10.1371/journal.pone.0169652 (PMC5215907; doi:10.1371/journal.pone.0169652)
Supplement: S3 Fig — Estimated percentage of any purchases of antidepressant medication per year among those with (a) continuous long-term unemployment, (b) intermittent unemployment, (c) re-employment in the second (d) third or (e) fourth year since the year of onset, as well as (f) the employed reference group. Adjusted for age, gender, education, living arrangements, and calendar year. (PDF) [file pone.0169652.s003.pdf]

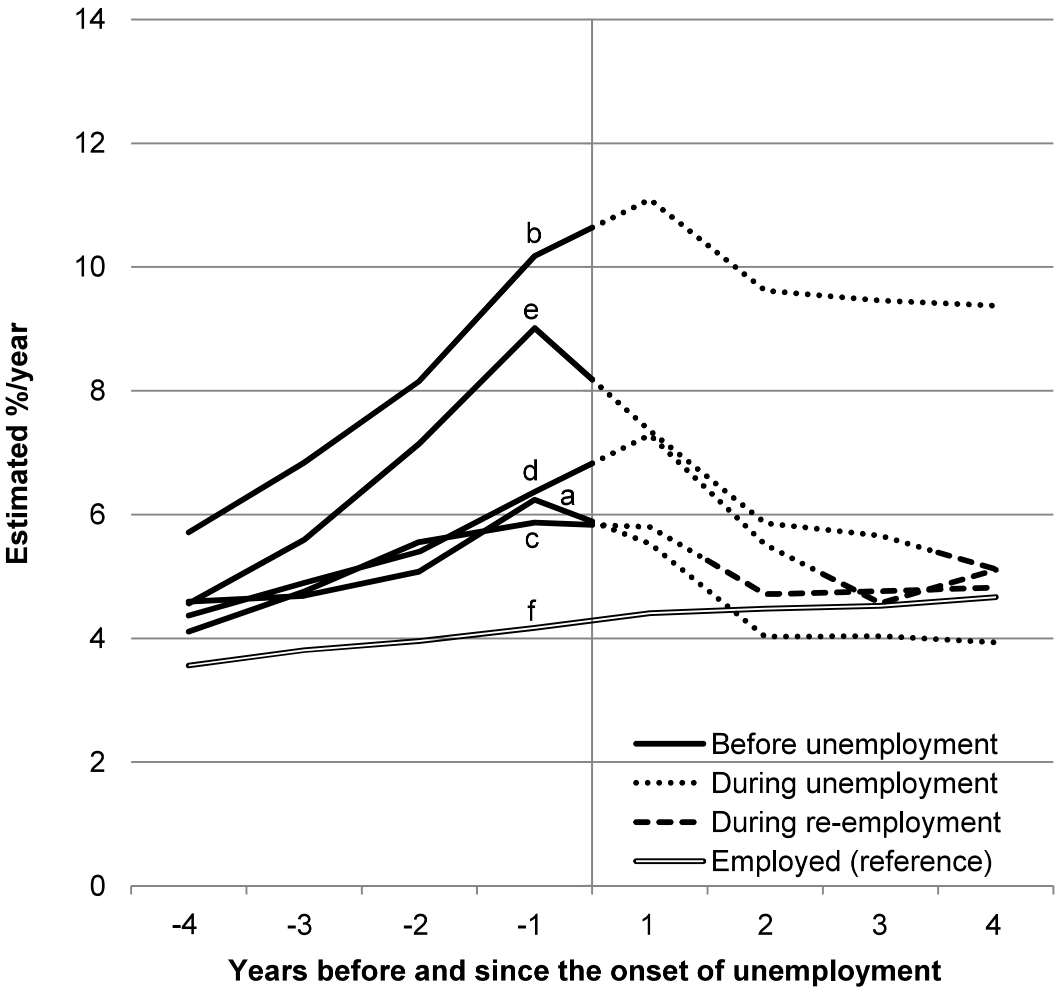

S3 Fig. Trajectories of antidepressant medication before and since the onset of unemployment using a dichotomous outcome measure. Estimated percentage of any purchases of antidepressant medication per year among those with (a) continuous long-term unemployment, (b) intermittent unemployment, (c) re-employment in the second (d) third or (e) fourth year since the year of onset, as well as (f) the employed reference group. Adjusted for age, gender, education, living arrangements, and calendar year.
